# A Case of Hepatitis in Pregnancy

**DOI:** 10.7759/cureus.75861

**Published:** 2024-12-17

**Authors:** Roslyn Alfred-Demas, Hubert Daisley, Nicola Dalrymple, Renee Alfred, Akilah Thompson-Dillon

**Affiliations:** 1 Internal Medicine, Tobago Regional Health Authority, Scarborough, TTO; 2 Pathology, Tobago Regional Health Authority, Scarborough, TTO; 3 Radiology, Tobago Regional Health Authority, Scarborough, TTO; 4 Pediatric Medicine, Tobago Regional Health Authority, Scarborough, TTO

**Keywords:** antibodies in liver disease, autoimmune disease in pregnancy, autoimmune hepatitis, hepatitis in pregnancy, icteric, jaundice, jaundice in pregancy, liver biopsy, liver disease in pregnancy, pregnancy

## Abstract

Autoimmune hepatitis (AIH) is rare, and the diagnosis is not always clear-cut. It is associated with increased morbidity and mortality for the pregnant mother and fetus. Frequent pregnancy testing of women in childbearing years who have a known diagnosis of AIH may be necessary, even after counseling on family planning takes place. Close evaluation and a multidisciplinary approach are essential in pregnancy as disease sequelae can become progressive. We report an interesting case of hepatitis in pregnancy, with an eventful clinical history. The patient in hand was started on treatment with a positive response to steroids. The liver biopsy was not confirmatory, but the laboratory results and the clinical scenario pointed to autoimmune etiology. We wish to share relevant management lessons and highlight important caveats of learning for future clinical relevance, as well as the importance of a liver biopsy.

## Introduction

Jaundice in a patient warrants a thorough investigation, and in a young woman, autoimmune disease must be ruled out. Furthermore, jaundice in pregnancy heralds the need for promptness in achieving a diagnosis. Schaefer comments that hepatitis can be due to infectious and noninfectious causes that include drugs, alcohol, immunologic (autoimmune hepatitis (AIH), primary biliary cholangitis (PBC)), or secondary to biliary tract dysfunction (cholestasis), pregnancy-related liver dysfunction, or malignancy. Schaefer goes on to emphasize that a clinical and essential pearl to remember is eliciting a thorough clinical history [1]. Elevation of serum transaminase levels, such as aspartate aminotransferase (AST) and alanine aminotransferase (ALT), is suggestive of hepatocellular injury, while an increase in the levels of alkaline phosphatase (ALP) and gamma-glutamyl transferase (GGT) reflects underlying cholestasis. According to Braga, patients with autoimmune hepatitis may have multiple clinical presentations [2]. Various literature reiterates that a liver biopsy is indicated if the diagnosis is not clear and in patients presenting with atypical clinical features [1]. We report an interesting case of autoimmune hepatitis in pregnancy and present and share interesting findings. Pregnant patients with AIH experience a plethora of challenges. Preconception care and pregnancy management must be individually tailored by a multidisciplinary team [2].

## Case presentation

A 30-year-old woman presented with a six-week history of gradual onset of yellowing of her eyes and skin. This was the first episode. She was not known to have any previous chronic medical illness, except for polycystic ovaries, for which she was placed on metformin but discontinued when she noticed the yellowing. She reported that a week prior to the yellowing of her skin and eyes, she had generalized weakness accompanied by a non-productive and intermittent cough and rhinorrhea throughout the day and night. There was no fever or other respiratory symptoms and no myalgia or night sweats. She experienced pruritus and non-bilious, non-projectile vomiting with pale stools and dark urine. There was no nausea, headaches, diarrhea, or blood in the stools. There were no neurological symptoms. She had no prior history of foreign travel, intravenous drug use, herbal remedies, blood transfusions, recent tattoos, substance abuse, dry eyes or dry mouth, oral ulcers, diarrhea, constipation, abdominal pain, Raynaud’s phenomenon, arthralgia, photosensitivity, or any skin rashes. Her younger sister, who had type 1 diabetes mellitus, died at age 28 years of age from complications related to diabetic nephropathy and renal failure. Social history did not reveal cigarette use, but only occasional alcohol intake. She was a registered nurse by profession and lived in an apartment with all basic amenities and no rodents in the vicinity. On examination, she was emaciated, afebrile, and hemodynamically stable. No asterixis was seen, but she has pruritic marks and jaundiced mucous membranes. The cardiorespiratory examination was normal.

There were no peripheral stigmata of chronic liver disease, except hyperpigmentation, and no organomegaly on abdominal examination. Mental state examination and neurological examination were also normal. She was referred to the dietitian for nutritional advice.

**Figure 1:**
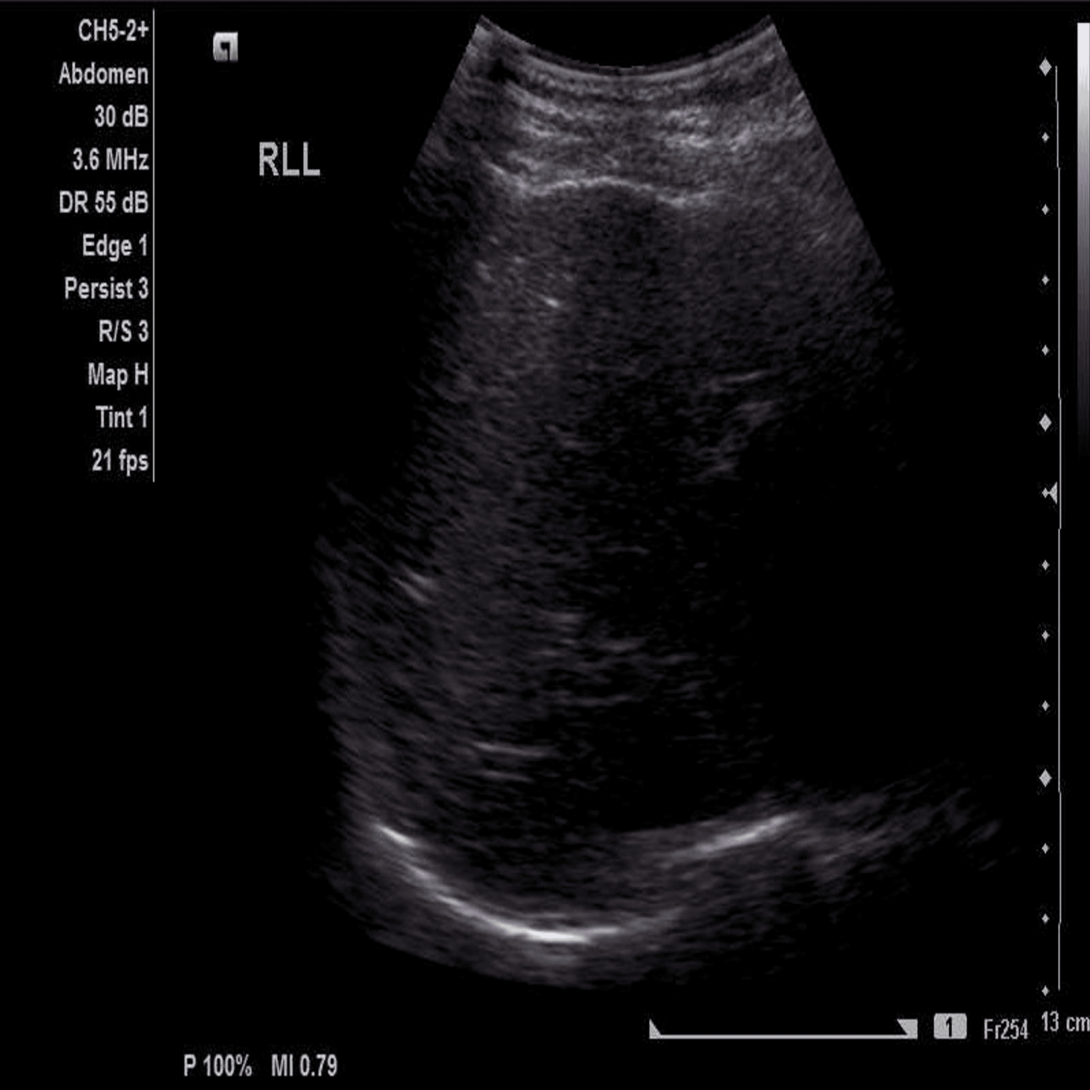
Ultrasound of the abdomen revealing normal liver size.

**Figure 2:**
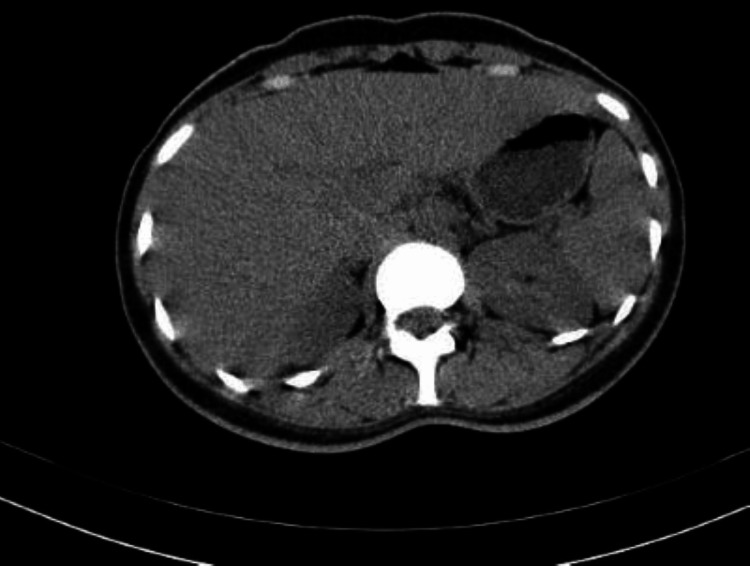
Computed tomography (CT) of the abdomen showing the liver and no evidence of biliary obstruction.

Initially, the urine pregnancy test was negative. Covid-19 PCR, along with HIV and hepatitis B and C serologies, were negative. General blood tests showed normocytic anemia with leukocytosis and normal renal function. Hyponatremia secondary to the vomiting was noted with a mildly elevated international normalized ratio (INR). She had direct hyperbilirubinemia, and all liver enzymes were severely deranged in a cholestatic pattern (Table 1). Immunoglobulin G levels and C-reactive protein levels were elevated. The immunology screen revealed a strongly positive antinuclear antibody (ANA) >1:160 titers, positive anti-smooth muscle antibody, and positive anti-Ro52 antibody testing. Under the antimitochondrial panel, the anti-LKM-1, anti-LC-1, and anti-SLA/LP were negative. Rheumatoid factor and dsDNA were negative, and iron and copper levels were normal. Serum ceruloplasmin levels were normal, and ophthalmological examination revealed the absence of Kayser-Fleischer rings. Hemoglobin electrophoresis, HbA1c, and thyroid function tests were normal. Upper gastrointestinal endoscopy showed no esophageal varices. The colonoscopy was normal. The breast ultrasound was normal. Ultrasound and computed tomography (CT) scans of the liver were normal, with liver size reported as normal with a normal gallbladder and no evidence of focal hepatic lesion. The ultrasound scan is depicted in Figure 1, and the CT scan is shown in Figure 2. She was assessed as having autoimmune hepatitis. Table 1 shows her laboratory results. 

**Table 1:** Trend of laboratory results. ALP: alkaline phosphatase; ALT: alanine aminotransferase; AST: aspartate aminotransferase; GGT: gamma-glutamyl transferase; INR: international normalised ratio; UPT: urine pregnancy test

Normal range	Month 1	Month 2 positive UPT	Month 3	Month 4	Month 5	Month 7	6 weeks postpartum
Hemoglobin g/l (12-15)	10	10.6	10.1	10.5	11.0	8.3	9.3
MCV fl mean corpuscular volume (83-110)	81	79	88.6	85	82.9	82	80
Platelet X 10 ^9^/l (150-410)	362	428	485	435	375	354	370
White blood cell x 10^9^/l (4-10)	4.4	18.2	14.3	13.5	15.9	11.8	10
Albumin g/dl (3.5-5.3)	4.1	3.2	3.2	3.3	3.6	3	2.9
ALP U/L (45-87)	495	853	1171	925	992	722	1457
GGT U/L (0-40)	60	923	1171	1056	1151	1093	2160
AST U/L (0-32)	311	250	290	310	323	154	252
ALT U/L (0-33)	277	305	377	431	534	180	368
Direct bilirubin mg/dl (0-0.2)	23.6	20.7	21.5	9.5	8.7	12.4	15.7
Total bilirubin mg/dl (0-1.2)	26.4	22.5	22.5	18.6	17.7	12.8	16.9
INR	1.5	1.5	1.6	1.3	1.1	1.1	1.2
Creatinine mg/dl(0.5-0.9)	0.8	0.6	0.7	0.6	0.5	0.4	0.5

After she completed a course of antibiotics, she was started on prednisolone (30 mg orally (PO) once daily (OD)) and azathioprine (50 mg once daily). A prompt referral to the gastroenterologist for further follow-up was done for a multidisciplinary approach. She was counseled on family planning methods, including barrier methods, and advised to avoid pregnancy until her liver disease was stable and better controlled.

Two months after treatment was started, the patient had a positive urine pregnancy test (UPT), and azathioprine with prednisolone was continued. She was promptly referred to the antenatal clinic, and a close monthly follow-up was arranged.

Four months into treatment, azathioprine was stopped due to worsening liver function tests, as shown in Table 1, as advised by the gastroenterologist. Although the serum thiopurine methyltransferase (TPMT) enzyme levels were normal, the team wanted to ensure that the azathioprine was not causing this response versus the pregnancy or failure of treatment. The prednisone dosage was increased to 60 mg once daily and tapered more slowly every four months, and a slight persistent improvement was noted in the liver transaminase levels (Table 1: month seven).

An MRI of the liver and a magnetic resonance cholangiopancreatography (MRCP) were booked for the second trimester, and no obstruction was noted. However, hepatomegaly was reported. The patient refused to have a liver biopsy during the second trimester, although it was offered. The pregnancy progressed and led to a healthy baby male, delivered via spontaneous vaginal delivery.

**Figure 3:**
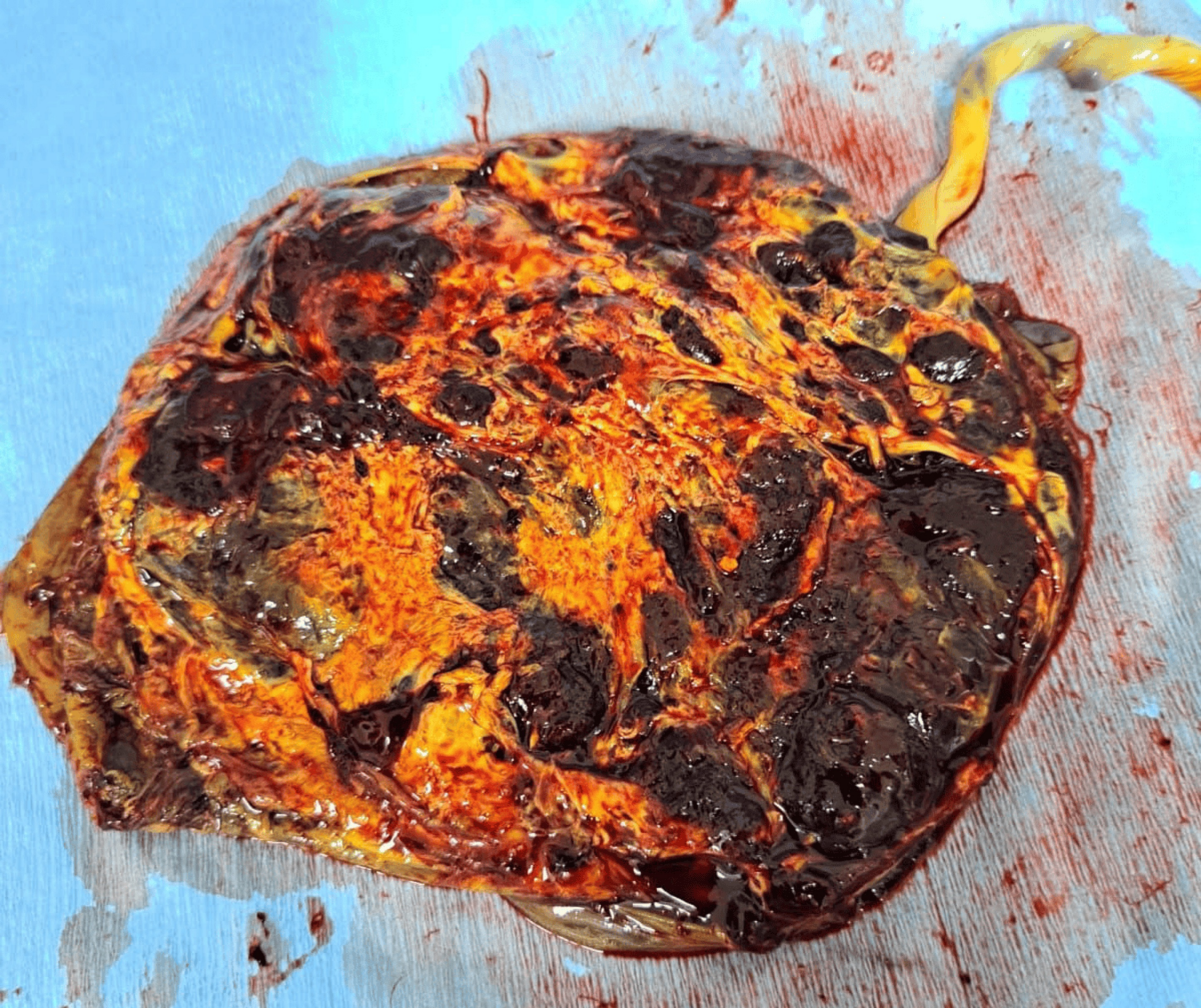
Placenta post-delivery showing areas of necrosis and jaundice.

Her placenta was icteric with areas of necrosis as noticed by the pediatrician on duty (Figure 3).

Six weeks after delivery, the liver transaminases increased, prednisolone was increased to 60 mg, and the patient was counseled on barrier methods for family planning. MRCP and MRI of the liver were repeated postpartum, and no focal lesion was seen. The liver function improved, and a liver biopsy was done ten (10) months postpartum (Table 1). Prednisone was tapered, and azathioprine was restarted. An ultrasound-guided liver biopsy was done under sterile conditions by the radiologist on duty.

Histology revealed feathery degeneration of hepatocytes with cholestasis in the biliary ductules. 

**Figure 4:**
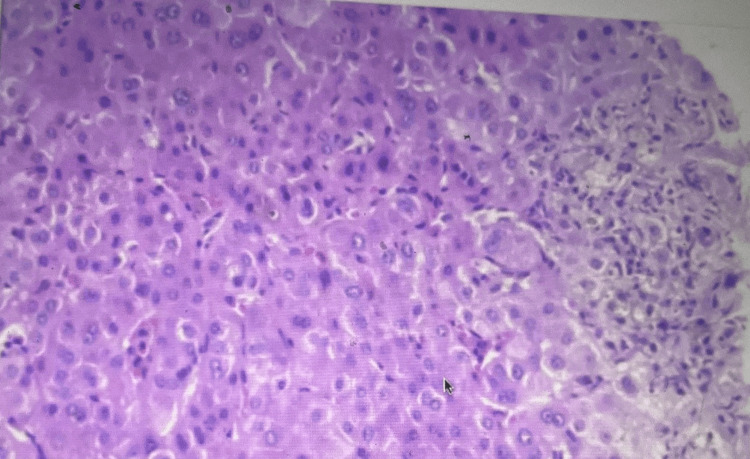
Liver histology sample for patient off medication.

**Figure 5:**
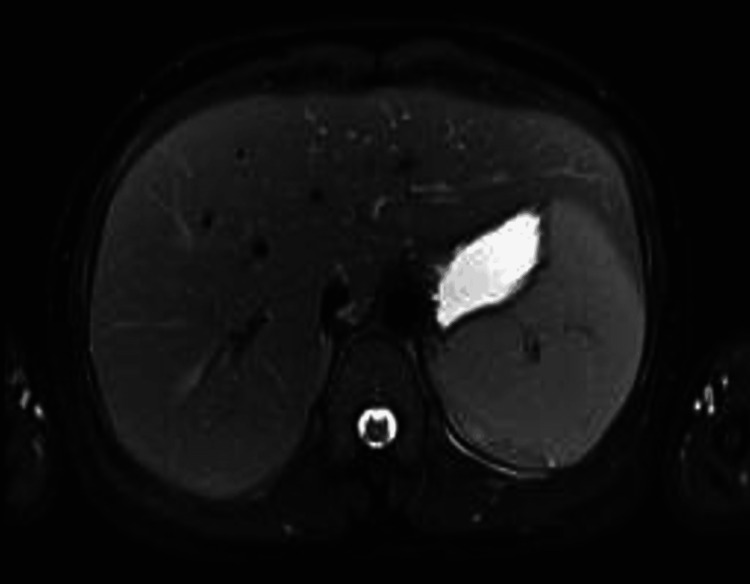
MRCP of the liver. MRCP: magnetic resonance cholangiopancreatography

A year later, another biopsy was done after being nonadherent to medications for three months (Figure 4). Histology returned showing periportal edema and ductular reaction in the portal tracts. Lobules showed conspicuous canalicular cholestasis and associated cholate stasis, features of long-standing cholestasis. The bile ducts were severely injured with biliary epithelial degeneration and intraepithelial lymphocytes. There were no granulomata significant lymphoid infiltrates, florid duct lesions, or features of primary biliary cholangitis. Interface hepatitis and a plasmacytic infiltrate, features of autoimmune hepatitis, are also absent. Steatosis and significant iron deposits were absent. There was no periportal fibrosis. A trichrome stain highlighted some onion skinning fibrosis around a bile duct. Overall, the features supported long-standing large bile duct obstruction, which, however, was not evident on repeat MRCP radiological imaging (Figure 5).

**Figure 6:**
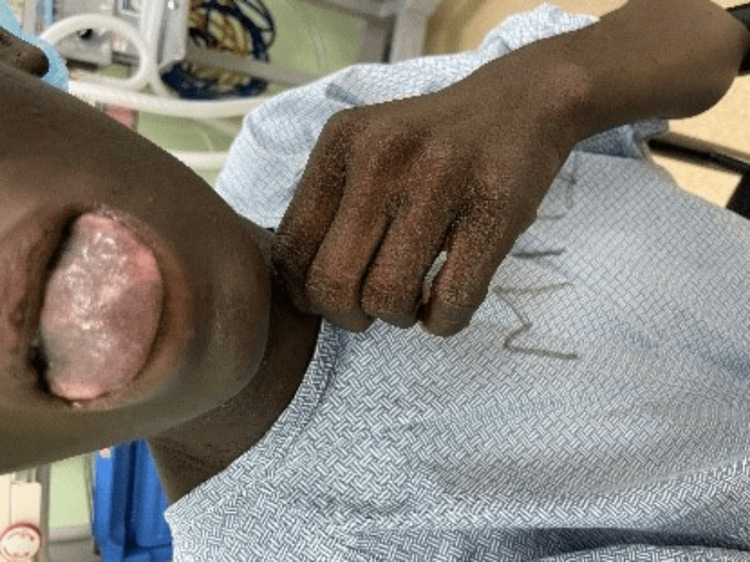
Hyperpigmented tongue and hand.

**Figure 7:**
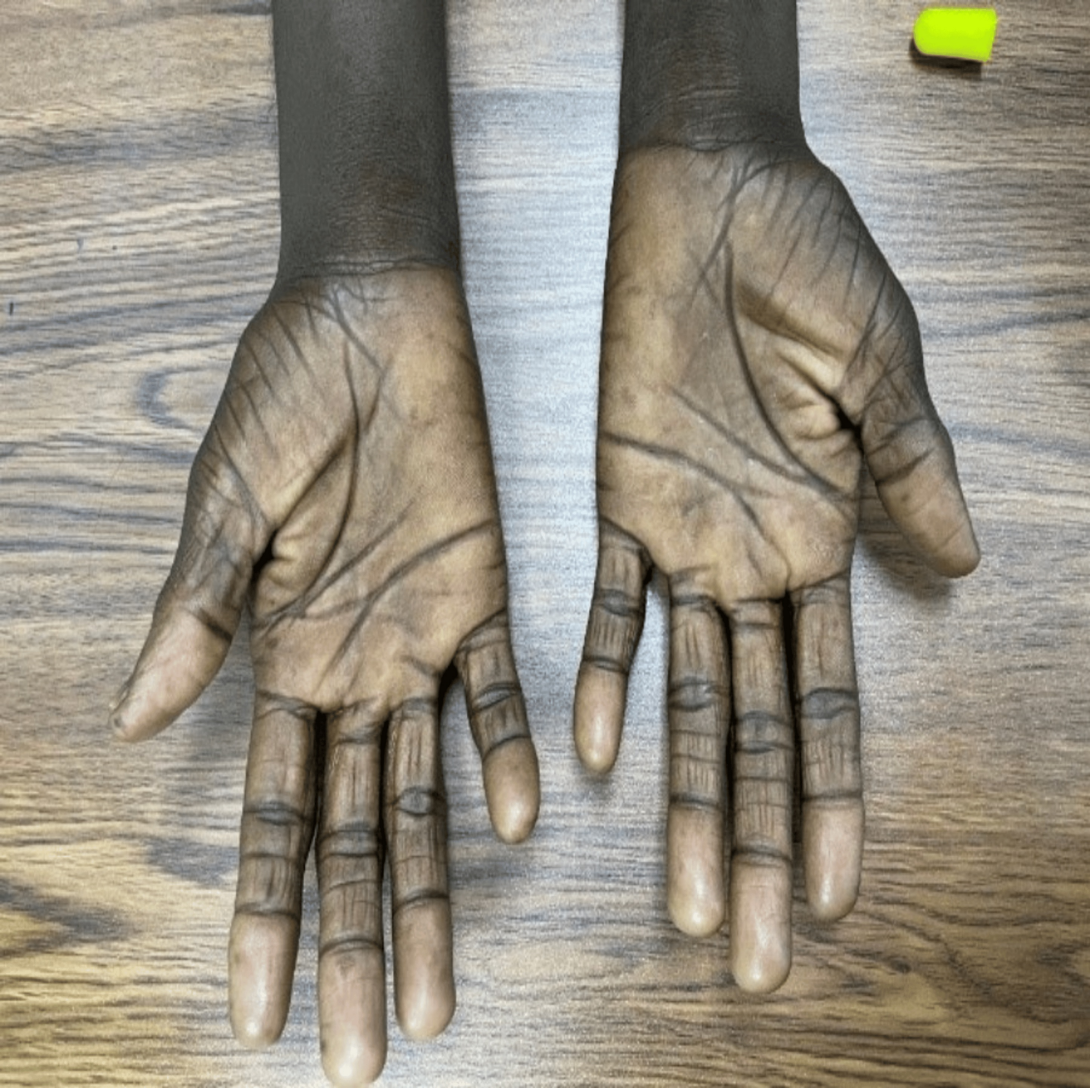
Hyperpigmented palms.

The patient became noncompliant with her medication, and her liver transaminases began to worsen, causing persistent hyperpigmentation and pruritus of her skin and weight loss, fatigue, and alopecia (Figures 6, 7). She was examined by the dermatologist, who pointed to the liver as the underlying cause of her hyperpigmentation, and no further therapy was offered. Hyponatremia was persistent, and repeat autoimmune screening additionally revealed positive SS-A and SS-B autoantibodies. Spot was urine sodium 12 mmol/L, and morning serum cortisol was 13.2 ug/dl, which was normal. Uric acid was normal. IgG remains elevated (34 g/L). Normal IgG range was reported as 7 to 16 g/L, and normal serum cortisol range was 4.8 to 19.5 ug/dl.

The Synacthen test was performed as part of the workup for hyperpigmentation and was normal. Adrenocorticotrophin (ACTH) levels and other pituitary hormone levels (follicle-stimulating hormone, luteinizing hormone, prolactin, and thyroid-stimulating hormone) were also normal. MRI brain and pituitary and CT chest were normal. As she had a history of prolonged steroid use, a DXA bone density scan was done routinely and also reported as normal.

Rifampicin was administered for pruritus. Ursodeoxycholic acid therapy was not available. Prednisolone and azathioprine were restarted after counseling on medication adherence was done. The next step will be to consider genetic testing and mycophenolate mofetil therapy if liver function worsens and continue family planning counseling with advice on barrier contraception.

## Discussion

This case poses a diagnostic dilemma as infectious causes were excluded, and she had a strongly positive autoimmune serology with an absence of drug or alcohol history. A positive response to steroids was observed. One wonders if the delay in the biopsy could have impacted the negative liver biopsy. The radiological investigations certainly did not lead to a diagnosis of biliary obstruction.

According to Schaefer [1], hepatitis is inflammation of the hepatic parenchyma or hepatocytes resulting in deranged liver enzymes [1]. If the period of inflammation or hepatocellular injury lasts for less than six months, characterized by normalization of the liver function tests, it is called acute hepatitis. In contrast, if the inflammation or hepatocellular injury persists beyond six months, it is termed chronic hepatitis.

We wish to highlight certain caveats of the disease entity. Braga stated that autoimmune hepatitis (AIH) is an immune-mediated illness that may lead to chronic and circuitous sequelae and presentation that is not always straightforward [2]. Recent research by Trivedi et al. has shown that it can affect all ages and all populations. Although rare, it must be considered in a young female who presents with jaundice, as in the case described [3].

A thorough evaluation is needed to exclude other causes of chronic liver disease, including infections, alcohol- or drug-induced, and hereditary or metabolic-associated steatosis etiology. Muratori et al. reported that while it is characterized by serum autoantibodies, distinct histological patterns aid the confirmation [4].

Like Czaja's and Domerecka's observations, the International Autoimmune Hepatitis Group (IAHG) has a diagnostic criterion that requires serum aspartate (AST) and alanine aminotransferase (ALT) abnormalities with elevated serum IgG levels and positive autoimmune serological investigations such as ANA, smooth muscle antibodies (SMA), and anti-LKM1 [5,6]. In the case discussed, these parameters were met, but the delay in histological diagnosis posed implications on the choice of treatment extension postpartum. IAHG has acknowledged that there is an autoantibody-negative phenotype (cryptogenic hepatitis) and that some patients who start with negative ANA and SMA eventually develop positivity. The element of overlap syndrome also necessitates the histological part of their criteria. The Paris criteria are used to aid with the diagnosis of the overlap syndrome of autoimmune hepatitis and primary biliary cirrhosis, where the presence of antimitochondrial antibodies and histology showing bile duct loss or injury is present. This patient’s elevated serum alkaline phosphatase was a cause for concern as it could be associated with PBC, although her imaging and biopsy did not indicate such. Her elevated IgG and positive response to steroids also suggested AIH.

A liver biopsy is mandatory even after positive autoimmune testing. The patient must be counseled on this fact. Even in this case, it was discussed in the first trimester, and the patient refused. Muratori’s article in the British Medical Journal states that the degree of inflammation and fibrosis cannot be reliably depicted by the transaminases and that to describe histology as typical of autoimmune hepatitis requires the presence of two out of three features: interface lymphocytic hepatitis, emperipolesis, and hepatocellular rosettes [4]. Even with this knowledge, Tiniakos conferred that recently IAHG proposed that liver biopsy can be considered as likely possible or unlikely AIH. Tiniakos also stated that no single histological finding is pathognomic for autoimmune hepatitis [7]. The delay in this patient’s biopsy may have caused an atypical histology pattern, hence the need to counsel patients for this to be done early in the course of the disease.

Frequent pregnancy testing may be needed in these cases, as ill medical patients may not have regular menstrual periods, and while no schedule is recommended, patients can be cautioned on barrier contraception and missed menstrual periods and symptoms of pregnancy so as to prompt pregnancy screening. Pregnancy should only be initiated in remission. Mack et al. also highlighted that treatment modalities can be teratogenic [8]. Close and frequent evaluation is needed in pregnancy as increased flares and exacerbations can be expected [2]. Liberal et al. and the European Association for the Study of the Liver (EASL) confirm that steroids and azathioprine are usually first-line, but other therapies exist [9].

According to Pape et al., the initial goal of treatment is the induction of a full biochemical response, defined as a normalization of both transaminase and IgG concentrations [10]. The European Association for the Study of the Liver states that treatment requires a multidisciplinary approach (gastroenterologist, immunologist, pathologist, radiologist, and nutritionist) [11].

The prognosis of this case is varied, as she is young, and it is believed that ethnicity of African descent can be associated with a poorer prognosis in AIH, using the guidelines from EASL [11].

## Conclusions

Lessons learned will be based on family planning counseling and multidisciplinary approaches to such cases. Contraception counseling is essential in such cases, and being mindful of repeating pregnancy tests when changing drug regimens is important. Psychiatric and psychological intervention can be used formally for counseling. Clinicians must always push for ongoing multidisciplinary meetings in chronic disease cases even when access to a gastroenterologist may be a limiting factor. Clinical correlation is important when intertwining radiology and histology with the facts of the case. Continuous review of the patient’s history and the beginning of long-standing cases can be useful.
